# Revising the Interpretation of Transcranial Doppler Ultrasound Examinations in Pediatric Cerebral Malaria

**DOI:** 10.4269/ajtmh.24-0332

**Published:** 2024-08-13

**Authors:** Nicole F. O’Brien, Taty Tshimanga

**Affiliations:** ^1^ Nationwide Children’s Hospital, The Ohio State University, Columbus, Ohio;; ^2^ Hopital Pediatrique de Kalembe Lembe, Universite de Kinshasa, Kinshasa, Democratic Republic of the Congo

## Abstract

Cerebral malaria (CM) is a devastating disease globally. Transcranial Doppler ultrasound (TCD) has identified five different phenotypes of deranged cerebrovascular hemodynamics in children with CM, each associated with different outcomes. For TCD to be used as a point of care neurodiagnostic and neuromonitoring tool in CM patients, proper interpretation of examinations is paramount. Comparison of measured cerebral blood flow velocities (CBFVs) to age-matched normative values is needed to interpret any pediatric TCD study. Until recently, normative values in African children did not exist, so previous work reported the frequency of CM phenotypes by classifying studies compared with normative values of European children. Now that normative TCD values in healthy African children have been established, we performed this retrospective analysis of prospectively collected data to determine phenotype frequency and associated outcomes in children with CM by comparing CBFV values to these contemporary controls.

## INTRODUCTION

Malaria is a significant global scourge with more than 249 million cases reported in 2022, an increase of 16 million cases from 2019. Malaria-related deaths are also on the rise, up to 608,000 from 576,000 in that same period. Cerebral malaria (CM) is a severe form of the disease with case fatality rates ranging from 15% to 40%. Long-term neurodisabilities, including cognitive deficits, attention and behavioral issues, spastic quadriplegia, and epilepsy, have been described in up to 50% of survivors.^1^ Additional insight into key pathophysiologic contributors to neurologic injury in these children is needed to develop adjunctive therapies that one day can improve outcomes.

Transcranial Doppler ultrasound (TCD) is a portable, noninvasive tool that readily evaluates cerebral blood flow velocities (CBFVs) in all the major vessels of the circle of Willis. The technique is repeatable, with interpretation available in real time, making it an ideal “stethoscope into the brain.” As such, we used TCD as a point of care measure of the underlying cerebrovascular hemodynamics in several cohorts of African children with CM.^2^^,^^3^ Five distinct alterations to TCD CBFVs and morphologic waveforms have been identified. These derangements, or phenotypes, include hyperemia, isolated posterior high flow (IPH), low flow, vasospasm, and microvascular occlusion (MO). As each group has prognostic utility and may also inform treatment decisions in children with CM, appropriate phenotypic classification is imperative.

One major consideration when utilizing TCD in pediatrics is the need to evaluate the measured CBFVs compared with age-matched children. To meet the metabolic demand of rapid brain growth in early childhood, cerebral blood flow increases significantly from birth through approximately age 6 years and then declines to adult levels by adolescence. Thus, a TCD examination can only be considered normal or abnormal if CBFVs are outside the expected range for a healthy child of the same age.

In the absence of any normative data for African children, we previously followed international consensus recommendations and, in previous manuscripts describing TCD phenotypes in children with CM, interpreted examinations by comparing them to normative data published by Bode and Eden^4^^,^^5^ in 1988, a cohort of healthy, European children. When a flow velocity was > or <2 SDs from these normative values, it was considered abnormal. We recently published normative TCD values for a large, healthy population of Congolese, Zambian, and Malawian children, where we identified significantly lower CBFVs in nearly every cerebral vessel in all age groups ≥3 years compared with those of Bode and Eden.^6^

Given this finding, we hypothesized that fewer children with CM have a true low flow phenotype and more have a normal TCD than previously reported. Furthermore, children previously classified into the hyperemia phenotype likely included only those with the most significant flow elevations, so under-classification into this phenotype also likely occurred. We therefore performed this retrospective review and report the frequencies of each phenotype when comparing CBFV measurements to two published control groups including healthy European children and healthy African children, where 2 SDs from these values was used to categorize flow as abnormal. Given work that has shown flow alterations > or <1 SD may be clinically relevant in central nervous system infections, we also report the rates of each phenotype using this lower threshold as a cutoff value for abnormal.^7^ Finally, we explored the relationships between the assigned phenotypes and outcomes.

## MATERIALS AND METHODS

### Patient population and general care of CM patients.

This study occurred at Queen Elizabeth Central Hospital in Blantyre, Malawi, at Kalembe Lembe Hospital in Kinshasa, Democratic Republic of the Congo (DRC), and at Lodja District Hospital in Lodja, DRC from January 2021 to May 2024. Ethics approval was obtained in each country (DRC CES ID 79/CNES/BN/PMMF/2021, Zambia ERES ID 00005948, Malawi COMREC P.02/21/3270). The cohort of children with CM presented here is unique from others that our group has previously published on, but they were recruited from the same sites throughout sub-Saharan Africa.

Parents or guardians of children 6 months to 12 years of age who met the WHO case definition of CM (*Plasmodium falciparum* parasitemia, Blantyre Coma Score ≤2, and no other discernable cause of encephalopathy) were approached for enrollment and provided informed consent. Children with known developmental delay and sickle cell disease were excluded, given the high frequency of abnormal TCD examinations in these populations. Likewise, given the unknown impact of severe malnutrition (mid-upper arm circumference <11 cm) or advanced HIV disease on TCD examinations, these children also were excluded.

Demographic data, vital signs, and physical examination findings were collected. Finger-prick samples were analyzed to determine parasite species and density, packed-cell volume, and blood glucose and lactate concentrations (Aviva Accu-Check, Zurich, Switzerland, and Arkray Lactate Pro 2, Kyoto, Japan). An admission lumbar puncture was performed, and the cerebrospinal fluid was analyzed for cell counts and chemistry. All patients received intravenous artesunate.

### Transcranial Doppler ultrasound examination.

Transcranial Doppler ultrasound was performed using a commercially available unit (NovaSignal, Los Angeles, CA). within 4 hours of admission. Middle cerebral arteries (MCAs), extracranial internal carotid arteries (Ex-ICAs), and basilar arteries were insonated using previously described methods.^8^ Systolic (Vs), diastolic (Vd), and mean flow (Vm) velocities were recorded at each interval. Pulsatility index (Vs – Vd/Vm) was automatically calculated by the TCD unit at each depth in each vessel. To differentiate causes of high CBFV values, the Lindegaard ratio (MCA Vm/Ex-ICA Vm) was calculated.

Based on admission TCD findings, participants were classified into one of the following phenotypes: normal, hyperemia, IPH, low flow, vasospasm, or MO. Table 1 outlines the diagnostic criteria where flow velocities in each vessel for each child with CM were compared with age-matched healthy control patients from both the old (European children) and new (African children) normative values. We also loosened the criteria to determine the frequency of each phenotype when considering flows ≥ or ≤1 SD from age normative value to be abnormal (compared with ≥ or ≤2 SDs as is noted in Table 1).

**Table 1:** Definitions used to categorize participants into transcranial Doppler ultrasound phenotypes

Normal Flow
Systolic, Diastolic, and Mean Flow Velocity in the Middle Cerebral Artery ±2 SDs from the Age Normal Value
Hyperemia
Systolic, Diastolic, and Mean Flow Velocity in the Middle Cerebral Artery ≥2 SDs above the Age Normal Value AND
LR* <3
Isolated Posterior High Flow
Mean Flow Velocity in the Basilar Artery ≥2 SD above the Age Normal Value AND
Mean Flow Velocity in Both Middle Cerebral Arteries within 2 SDs of the Age Normal Value
Low Flow
Systolic, Diastolic, and Mean Flow Velocity in the Middle Cerebral Artery ≤2 SDs below the Age Normal Value AND
PI^†^ <1.2
Vasospasm
Mean Flow Velocity in the Middle Cerebral Artery ≥2 SDs above the Age Normal Value AND
LR ≥3
Microvascular Obstruction/Alteration
Systolic Flow Velocity in the Middle Cerebral Artery within 2 SDs of the Age Normal Value AND
Diastolic Flow Velocity in the Middle Cerebral Artery ≤2 SDs below the Age Normal Value AND
PI ≥1.2

LR = Lindegaard ratio; PI = Pulsatility index.

* LR = mean flow velocity in the middle cerebral artery/mean flow velocity in the extracranial carotid artery.

^†^ PI = (systolic flow velocity − diastolic flow velocity/mean flow velocity).

Transcranial Doppler ultrasound studies were performed by individuals who had successfully completed the sub-Saharan African TCD Academy course content, which includes: 1) 12 online didactic lectures designed to teach basic concepts of TCD science including neuroanatomy, scan techniques, Doppler waveform characteristics, diagnostic criteria, and clinical applications in children; 2) 100 case studies aimed at developing interpretation skills; 3) a hands-on introduction to TCD training session to develop TCD scanning proficiency; and 4) a written examination. The course was considered successfully finished when didactics and case studies were reviewed, the participant demonstrated proficiency in TCD scanning (each measurement of a complete TCD examination had a coefficient of variation <10% from that of the trainer (NF O’Brien), and the written examination was passed. These steps were required to standardize acquisition of all TCD examinations by different practitioners.

### Outcomes.

The Pediatric Cerebral Performance Category (PCPC) measures and quantifies morbidity after pediatric critical illness.^9^ Scores range from 1 to 6, with 1 being a normal functional level and 6 being death. Other values represent progressive impairment: 2 = mild disability (alert and able to interact at an age-appropriate level but with mild cognitive, behavioral, or neurologic deficits), 3 = moderate disability (alert and able to carry out age-appropriate activities of daily life but with obvious cognitive or neurologic deficits that limit function), 4 = severe disability (conscious but dependent on others for all daily functions), and 5 = vegetative state (any degree of coma or an inability to interact with the environment). The PCPC was scored at the time of hospital discharge. Children with a PCPC of 1 or 2 were considered to have a good outcome, whereas those with a PCPC of 3–5 were considered to have survived with moderate to severe disability. Participants who died were scored as a 6.

## STATISTICAL ANALYSES

Demographic and laboratory variables were summarized using medians with interquartile ranges, means and SDs, and frequencies with percentages where appropriate. Differences between previously calculated phenotype frequencies and currently calculated phenotype frequencies were assessed using χ^2^ tests. To evaluate the probability of outcome (good versus bad) differing by phenotype, multinomial regression determined odds ratios and 95% CIs. All analyses were conducted using GraphPad Prism.

## RESULTS

**Figure 1.**
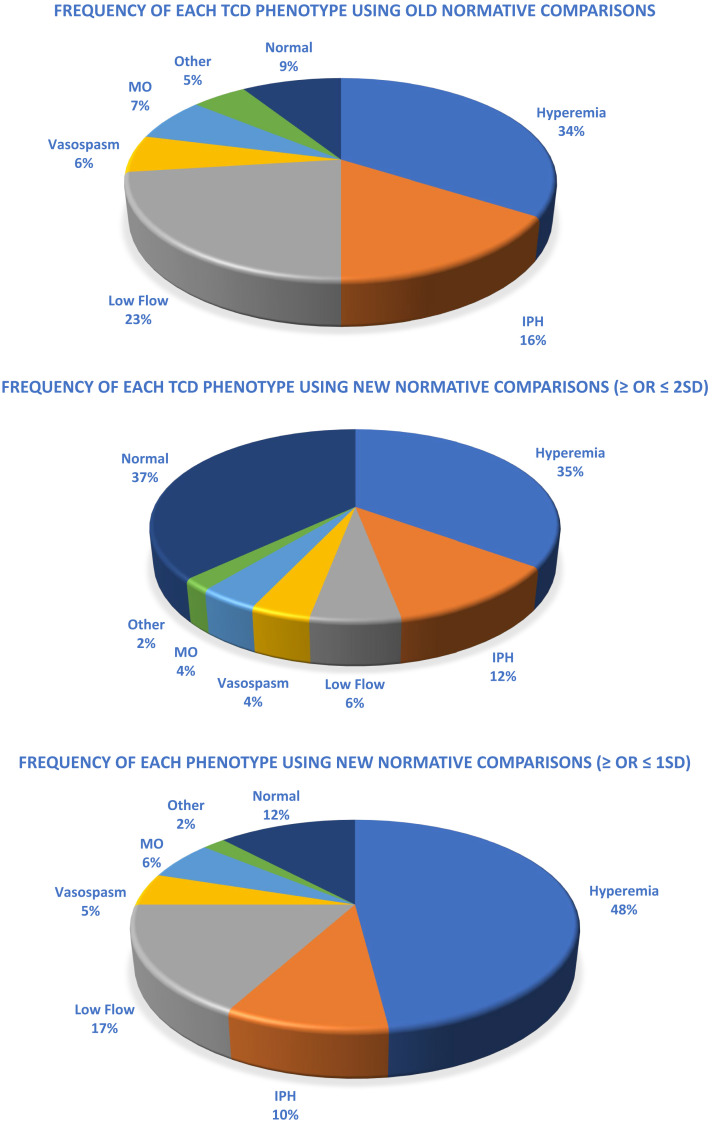
The frequency of each TCD phenotype in children with cerebral malaria when interpreting results compared with age-matched healthy European children and when interpreting results compared with age-matched healthy African children (both > or <2 SDs and > or <1 SD from age normal). IPH = isolated posterior high flow; MO = microvascular occlusion; TCD = transcranial Doppler ultrasound.

Retrospective analysis of 280 sequentially prospectively enrolled patients was performed. Demographics for the cohort are presented in Table 2. When interpreting this cohort’s TCD results by comparing them to age-matched healthy European children, we identified a normal TCD examination in 9.2% (*n* = 25). The rest of the studies were classified as hyperemia: 34% (*n* = 95); IPH: 16% (*n* = 45); low flow: 23% (*n* = 64); vasospasm: 6% (*n* = 17); and MO: 7% (*n* = 20). Fourteen (5%) studies were classified as other/mixed-flow phenotype (Figure 1).

**Table 2:** Cohort demographics (*N* = 280)

Variable	Value
Demographics	
Age (months), Mean (SD)	60 (±35)
Male, *n* (%)	145 (52)
Vital Signs	
Temperature (°C), Median [IQR]	37.3 [36–38]
Heart Rate (beats/minute), Median [IQR]	125 [110–140]
RR (breaths/minute), Median [IQR]	30 [26–35]
Oxygen Saturation (%), Median [IQR]	98 [96–99]
SBP (mm Hg), Median [IQR]	101 [92–106]
DBP (mm Hg), Median [IQR]	58 [53–68]
MBP, Median [IQR]	74 [66–81]
Laboratory Investigations	
Packed Cell Volume (%), Median [IQR]	25 [21–29]
Glucose (mmol/L), Median [IQR]	5.8 [4.8–6.7]
Lactate (mmol/L), Median [IQR]	2.8 [1.7–4.7]
Parasites/Microliter Blood, Median [IQR]	255,000 [92,00–664,000]
* Pf*HRP2 (ng/mL), Median [IQR]	570 [215–1,335]
Clinical Features	
Blantyre Coma Score, *n* (%)	
0	35 (13)
1	99 (35)
2	146 (52)
Outcome	
Good, *n* (%)	
PCPC 1–2 (normal, mild disability)	188 (67)
Poor, *n* (%)	
PCPC 3–5 (moderate to severe disability)	53 (19)
PCPC 6 (died)	39 (14)

DBP = diastolic blood pressure; IQR = interquartile range; MBP = mean blood pressure; PCPC = Pediatric Cerebral Performance Category; *Pf*HRP2 = *Plasmodium falciparum* histidine rich protein 2; RR = respiratory rate; SBP = systolic blood pressure.

When utilizing healthy African children as age-matched comparisons and considering abnormal to be ≥ or ≤2 SDs from these values, 37% (*n* = 104) of children with CM had a normal TCD examination. Thirty-five percent (*n* = 98) had hyperemia; 12% (*n* = 34) had IPH; 6% (*n* = 17) had low flow; 4% (*n* = 11) had vasospasm; 4% (*n* = 11) had MO; and 2% (*n* = 5) had other/mixed-flow phenotype (Figure 1).

When abnormal was considered to be ≥ or ≤1 SD from age-matched African children, 12% (*n* = 34) had a normal TCD examination; 48% (*n* = 134) had hyperemia; 10% (*n* = 28) had IPH; 17% (*n* = 48) had low flow; 5% (*n* = 14) had vasospasm; 6% (*n* = 17) had MO; and 2% (*n* = 5) had another/mixed-flow phenotype (Figure 1).

Both the hyperemia and IPH phenotypes were associated with decreased odds of a poor neurologic outcome, whereas low flow and MO were associated with increased odds of poor neurologic outcome (Table 3). However, the strength of the associations changed depending on which normative group was used for comparison and the number of SDs from the mean normal that were considered abnormal.

**Table 3:** Predicted probabilities (with 95% CIs) of outcomes in children with cerebral malaria by transcranial Doppler ultrasound phenotype

Phenotype	Probability of Poor Outcome	*P*-Value
Utilizing > or <2 SDs from Previous Normative Values		
Hyperemia	0.71 (0.41–1.3)	0.24
IPH	0.36 (0.15–0.89)	0.03
Low flow	1.9 (0.99–2.9)	0.08
Vasospasm	2.1 (0.78–6.1)	0.14
Microvascular Occlusion	2.8 (1.1–7.8)	0.04
Utilizing > or <2 SDs from African Normative Values		
Hyperemia	0.55 (0.32–0.98)	0.03
IPH	0.46 (0.27–0.9)	0.04
Low flow	5.8 (1.7–20.1)	0.005
Vasospasm	2.8 (0.88–9.0)	0.08
Microvascular Occlusion	1.2 (1.0–5.5)	0.04
Utilizing > or <1 SD from African Normative Values		
Hyperemia	0.51 (0.29–0.87)	0.01
IPH	0.47 (0.17–0.99)	0.03
Low flow	2.1 (1.0–3.4)	0.04
Vasospasm	2.1 (0.74–6.6)	0.16
Microvascular Occlusion	2.6 (1.1–6.7)	0.04

IPH = isolated posterior high flow.

## DISCUSSION

Transcranial Doppler ultrasound has identified five different phenotypes of altered CBFVs in children with CM. As biomarkers of underlying pathophysiology that can be used for prognostication and that may one day drive individualized intervention, proper phenotypic classification is imperative. Given newly published normative TCD values for African children that identified significantly lower measured CBFVs in this population, we reevaluated the frequency of each phenotype when interpreting results compared with these lower normative values. We also assessed the relationships between the classified phenotypes and neurologic outcomes.

We found that when considering values <2 SDs from those of healthy African children as abnormal, there were fewer children with CM with the low flow phenotype and more children with a normal TCD. When classifying values <1 SD from expected as abnormal, the rate of low flow phenotype identification continued to be slightly lower and normal TCD classification slightly more common than when using old comparison data. These findings are expected given the lower baseline values identified in healthy African children. Of note, although the low flow phenotype has been reported to be associated with poor outcome, strengthening of the association was noted when classifying a study as such compared with African normative data (odds ratio [OR]: 2.1, 95% CI: 1.0–3.4 if flows were <1 SD and OR: 5.8, 95% CI: 1.7–20.1 if flows were <2 SDs). This finding is likely explained by improved sensitivity of the classification approach when using appropriate normative values as a comparison. In this situation, when the low flow phenotype was identified, it represented significant and progressive reduction in substrate delivery to the tissue, which would increase the risk and severity of neurologic injury.

The hyperemia phenotype became more common and the IPH phenotype less common when interpretation of TCD examination results were compared with African normative values. When >2 SDs were used as abnormal, the frequency of hyperemia was 35%, and it increased to 48% when >1 SD was used, whereas IPH rates fell from 12% to 10%. This finding suggests that IPH and hyperemia may be a continuum of the same underlying pathophysiologic condition. A vasodilatory response due to malarial infection and neuroinflammation may first be identified in the posterior circulation given its greater density of parasympathetic fibers compared with other regions of the cerebral circulation. Vasodilation then progresses to the anterior circulation and becomes more generalized as stimulators of the response increase over time or duration of illness.^10^ This relationship is likely to have been uncovered because of the increased sensitivity of classification of phenotypes using the current approach. In addition, both hyperemia and IPH have been shown to be associated with lower odds of a poor outcome. That association became more pronounced when new normative values were utilized for phenotype classification. Using the old comparison values, to be classified as having hyperemia or IPH, cerebral blood flow would actually have been more than 1–2 SDs higher than normal and may have been as high as 4–5 SDs higher. Thus, although identification of a high flow phenotype is associated with improved outcomes, this relationship is most clear when hyperemia is mild to moderate (1–2 SDs above normal) and may become less pronounced or even be lost when very significant elevations in cerebral blood flow occur. This likely occurs owing to progressive increases in brain blood volume and intracranial hypertension.

Overall, given the findings presented above, we believe that TCD measurements in children with CM should be compared with the published normative data for African children for interpretation. Furthermore, given the clear associations with outcomes, we suggest that > or <1 SD should be considered abnormal and used for phenotype determination in this population. In addition, within a phenotype, the number of SDs from the normative values should be considered to further quantify the degree of flow derangement and aid in prognostication.

One limitation of the work is that advanced neuroimaging techniques such as computed tomography perfusion and magnetic resonance perfusion, which are considered the gold standard for measuring cerebral blood flow, were not available. Therefore, it was not possible to determine the true sensitivity of the described TCD measures as they relate to changes in cerebral blood flow in this population. However, TCD-derived CBFVs are close surrogates for cerebral blood flow if the diameter of the measured vessel does not change during the course of the investigation, something physiologically unlikely. In addition, although the signal toward associations with outcomes has been identified in this study, larger studies with longer follow-up of outcomes are needed to determine if alterations to measured CBFVs, particularly at the lower threshold of > or <1 SD from normal, represent clinically significant changes that, if intervened upon, would result in changes in outcome.

## CONCLUSION

When CBFVs in pediatric CM patients were compared with normative values in African children, the frequency of each TCD-derived phenotype was significantly different than previously described. Future studies utilizing TCD in children with CM should interpret examinations and assign phenotypes based on these new normative values.
